# Operational Optimization of Large-Scale Parallel-Unit SWRO Desalination Plant Using Differential Evolution Algorithm

**DOI:** 10.1155/2014/584068

**Published:** 2014-02-17

**Authors:** Jian Wang, Xiaolong Wang, Aipeng Jiang, Shu Jiangzhou, Ping Li

**Affiliations:** ^1^School of Aeronautics and Astronautics, Zhejiang University, Hangzhou 310027, China; ^2^Institute of Energy Utilization & Automation, Hangzhou Dianzi University, Hangzhou 310018, China

## Abstract

A large-scale parallel-unit seawater reverse osmosis desalination plant contains many reverse osmosis (RO) units. If the operating conditions change, these RO units will not work at the optimal design points which are computed before the plant is built. The operational optimization problem (OOP) of the plant is to find out a scheduling of operation to minimize the total running cost when the change happens. In this paper, the OOP is modelled as a mixed-integer nonlinear programming problem. A two-stage differential evolution algorithm is proposed to solve this OOP. Experimental results show that the proposed method is satisfactory in solution quality.

## 1. Introduction

The shortage of fresh water has become a bottleneck of the economic development in many countries. Seawater desalination is an effective way to solve this problem. Reverse osmosis (RO) desalinating is one of the most popular ways to generate freshwater from seawater and has made a rapid progress over the past four decades [1–3]. The scale of seawater reverse osmosis (SWRO) desalination plant is continually expanding, whose capacity of freshwater has exceeded 100,000 tons per day in recent years.

Now, a kind of large-scale parallel-unit SWRO desalination plant, which is composed of multiple parallel RO units, has appeared. This kind of plant has huger capital cost and more complicated operation processes. So, before it is built, an optimal design is made to select the suitable devices and system performance to match the operating condition [4–8]. These optimal designs are made based on static condition [9, 10], but the actual situation is changing [11]. For example, the seawater temperature varies with changing seasons, the freshwater supply changes according to the user's demand, the permeate rate is declining with the membrane fouling [12], and so on. The result is that these devices would not work at the optimal designed points in most time. Therefore, an operational optimization scheduling is necessary to make these machines work in a best way under the changed conditions.

In this paper, a mathematical model of operational optimization problem (OOP) for a large-scale parallel-unit SWRO desalination plant, which includes objective function and constraint functions, is made. In order to solve this OOP, a two-stage differential evolution (TSDE) algorithm is proposed. The simulating results show that the TSDE is excellent in searching ability than basic DE and genetic algorithm (GA).

## 2. Problem Description and Formulation

### 2.1. SWRO Desalination Plant Representation

**Figure 1:**
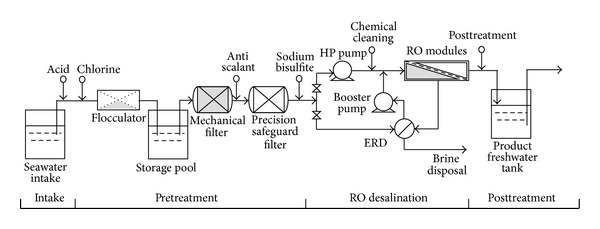
Schematic diagram of a single SWRO desalination unit.

The single SWRO desalination unit is a multistage process, which includes seawater intake, pretreatment, RO desalination, and posttreatment [13].  Figure 1 shows the schematic diagram of the unit.

In the intake stage, the raw seawater is pumped from a deep well, which is located close to the shoreline, to the flocculators to filter; in the pretreatment stage, the most suspended matters and colloids are filtered out of seawater by flocculators, mechanical filters, and precision safeguard filters successively to ensure that SDI (silt density index) is lower than 5 to meet the RO modules requirement; in the RO desalination stage, one part of the fed seawater is pressurized by the high pressure (HP) pump and the other by energy recovery device (ERD) and booster pump up to 40 ~ 50 bar and then is desalinized by RO modules; in the posttreatment stage, the produced freshwater flows into the product freshwater tanks (PFWT) to supply into the municipal water network; the brine is disposed reasonably.

**Figure 2:**
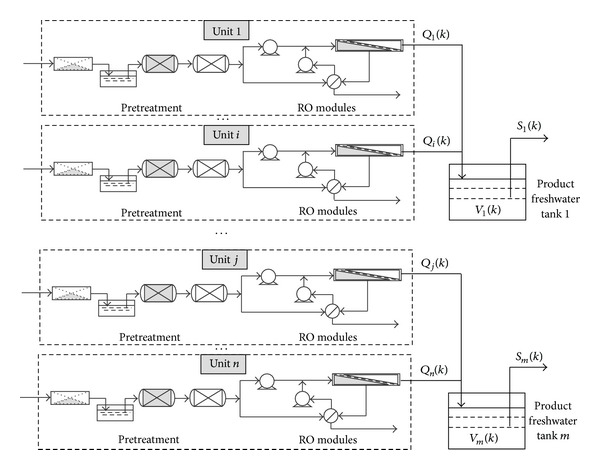
Schematic diagram of a parallel-unit SWRO desalination plant.

The schematic diagram of a large-scale parallel-unit SWRO desalination plant is shown in Figure 2, which is structured with a number of independent RO units in parallel. Each RO unit has different permeate rate. The freshwater, produced by RO Unit *i*  (*i* ∈ *n*_*j*_), flows into PFWT *j* and then is supplied for user.

### 2.2. Problem Mathematical Formulation

The OOP of a large-scale SWRO desalination plant is to make an optimal scheduling plan to minimize the plant's total running cost (TRC). This optimal schedule plan determines the on/off status and amount of generated freshwater of each RO unit, and the amount of supplied freshwater by each PFWT at each time period. When computing the TRC, the price factors, such as time-of-use electricity price, the operation cost, and the maintenance cost, are taken into consideration.

#### 2.2.1. Objective Function of OOP

A lumped parameter model of this problem is built in this paper. The TRC of the plant consists of capital depreciation cost (CDC), operating cost (OC), labor and chemical cost (LCC), and energy cost (EC), which is presented as

(1) $$\begin{array}{c} \min\;\;\text{TRC}=\text{CDC}+\text{OC}+\text{LCC}+\text{EC}. \end{array}$$

*(1)  The Capital Depreciation Cost, CDC, is*

(2) $$\begin{array}{c} \text{CDC}=\text{CC}\times 1.411\times \eta \times T. \end{array}$$

The CDC is presented as (2), where CC is the capital cost of the SWRO desalination plant, 1.411 is the coefficient that is used to calculate the practical investment, *η* is the capital charge rate, which is usually a constant, and *T* is the operational optimization periods. When the SWRO desalination plant has been built, the CC is a fixed value; therefore, the CDC is a fixed cost in a certain time period *T* under a constant capital charge rate *η*. So, when computing the minimum value of TRC in this condition, this part cost CDC can be ignored. When the other parts of TRC reach their minimum values, the TRC gets its optimal value by adding this constant cost.

*(2)  The Operating Cost, OC.* The OC includes maintenance expense and the repair and replacement expense of devices. The maintenance expense refers to the conventional maintenance expense during machines running; the repair and replacement expense refers to the cost of repair and replacement when the equipment stops. The OC is presented as

(3) $$\begin{array}{c} \text{OC}=\sum_{k=1}^{K}\sum_{i=1}^{n}M_{i}\left(k\right), \end{array}$$

where *K* is the time horizon of the operational optimization; *n* is the number of RO units in the plant; *M*_*i*_(*k*) is the operating cost of Unit *i* at the time *k*, which is presented as

(4) $$\begin{array}{c} M_{i}\left(k\right)=\{\begin{array}{cc} C_{1}\times Q_{i}\left(k\right), & \alpha_{i}\left(k\right)=1,\\ C_{2}, & \alpha_{i}\left(k\right)=0, \end{array} \end{array}$$

where *C*_1_ is the coefficient that is used to calculate the maintenance cost; *Q*_*i*_(*k*) is the amount of freshwater generated by Unit *i* at time *k*; *C*_2_ is the repair and replacement expense when Unit *i* stops at time *k*; *α*_*i*_(*k*) is the on/off status of Unit *i* at time *k*; *α*_*i*_(*k*) = 1 when Unit *i* is running; *α*_*i*_(*k*) = 0 when it stops.

*(3)  The Labor and Chemical Cost, LCC.* Usually, we consider LCC = 12% × TRC. This cost includes labor cost, chemical expense, and other expenses. When TRC is the smallest, LCC has its minimum value.

*(4)  The Energy Cost, EC.* The energy cost is the energy consumption which is necessary to drive the devices. It is obviously variable in different scheduling plans. By optimizing the scheduling plan, the plant's energy consumption can achieve the minimum value. The EC is presented as

(5) $$\begin{array}{c} \text{EC}=\sum_{k=1}^{K}\left(P_{e}\left(k\right)\sum_{i=1}^{n}\left(\alpha_{i}\left(k\right)\times C_{3}\times Q_{i}\left(k\right)\right)\right), \end{array}$$

where *C*_3_ is the coefficient between energy and amount of freshwater generated by Unit *i* at time *k*; *P*_*e*_(*k*) is time-of-use electricity price at time *k*.

#### 2.2.2. Constraints of OOP

The constraints of OPP include technical limitations and the design requirements, which are as follows.

*(1)  The Amount of Freshwater, Q*_*i*_(*k*).  *Q*_*i*_(*k*) is the amount of freshwater generated by Unit *i* at time *k*, which is subject to

(6) $$\begin{array}{c} Q_{i,\min}\leq Q_{i}\left(k\right)\leq Q_{i,\max},\;i=1,2,\ldots ,n,\;\;\forall k, \end{array}$$

where *Q*_*i*,max⁡_, *Q*_*i*,min⁡_ are the upper and the lower limit of the amount of freshwater generated by Unit *i*, respectively.

*(2) The Capacity of Each PFWT, V*_*j*_(*k*). *V*_*j*_(*k*) is the capacity of PFWT *j* at time *k*, which is subject to

(7) Vjmin⁡≤Vj(k−1)+∑i=1njαi(k)·Qi(k)−Sj(k)≤Vjmax⁡,           j=1,2,…,m;  i=1,2,…,ni,  ∀k,

where *V*_*j*_(*k* − 1) is the capacity of PFWT *j* at the beginning of time *k*; *m* is the number of PFWT; *n*_*j*_ is the number of RO units which feed freshwater to the PFWT *j*; *S*_*j*_(*k*) is the amount of supplied freshwater by PFWT *j* at time *k*; *V*_*j*,max⁡_, *V*_*j*,min⁡_ are the upper limit and the lower limit of capacity of PFWT *j*.

*(3)  The Amount of Supplied Freshwater, S*_*j*_(*k*). *S*_*j*_(*k*) is the amount of supplied freshwater by PFWT *j* at time *k*, which is subject to

(8) $$\begin{array}{c} \sum_{j=1}^{m}S_{j}\left(k\right)=D\left(k\right),\;\forall k,\\ S_{j}\left(k\right)\geq 0,\;j=1,2,\ldots ,m,\;\;\forall k. \end{array}$$

## 3. Two-Stage Differential Evolution

The OOP of the large-scale parallel-unit SWRO desalination plant is a mixed-integer nonlinear programming problem (MINLP) over the time horizon. In the OOP, {0, 1} binary variables represent the on/off statuses of RO units; the real variables represent the amounts of freshwater generated by RO units and supplied freshwater by PFWTs. In order to compute this OOP, a novel differential evolution, TSDE, is proposed in this paper, and the basic steps of this TSDE are addressed in the following subsections.

### 3.1. Basic DE

Differential evolution algorithm was originally proposed by Storn and Price for solving continuous optimization in the mid-1990s [14–18]. It is an evolutionary algorithm, including three important operators: mutation, crossover, and selection [19, 20]. The basic DE works are as follows.

**Step 1 (initialization)**

The original population is generated in the search space randomly, which contains NP individual vectors: **x**_*i*,1_ = [*x*_*i*1,1_, *x*_*i*2,1_,…, *x*_*iD*,1_], *i* = 1, 2, …, NP, where *D* is the dimension of individual.

**Step 2 (mutation)**

For each individual vector **x**_*i*,*G*_, a mutated solution **v**_*i*,*G*_ is created according to a DE/rand/1/bin mutate operator (9) in each generation *G* [15]. Consider

(9) $$\begin{array}{c} v_{i,G}^{}=x_{r_{1},G}+F\times \left(x_{r_{2},G}-x_{r_{3},G}\right), \end{array}$$

where *G* is the current generation number, *F* is a scale factor, and *r*_1_, *r*_2_, and *r*_3_ are randomly selected integers from the set {1, 2, …, NP} (*r*_1_ ≠ *r*_2_ ≠ *r*_3_ ≠ *i*).

**Step 3 (crossover)**

The crossover operator generates an offspring **u**_*i*,*G*_ according to

(10) uij,G={vij,G,if(rand(j)≤CR) or j=rnbr(j),xij,G,otherwise          (i=1,2,…,NP;j=1,2,…,D),

where CR is a crossover rate; rnbr(*j*) is a randomly chosen index (rnbr(*j*)∈{1,2,…, *D*}), which ensures **u**_*i*,*G*_ getting at least one component from **v**_*i*,*G*_.

**Step 4 (selection)**

The selection operator is to generate next population **x**_*i*,*G*+1_ according to (11). The objective function of **x**_*i*,*G*_ is compared to one of **u**_*i*,*G*_ and the smaller one is selected as the next generation. Consider

(11) xi,G+1={ui,G,if(f(ui,G)≤f(xi,G)),xi,G,otherwise             (i=1,2,…,NP).

**Step 5 (stopping criterion)**

If the stopping criterion (maximum number of iterations) is satisfied, computation is terminated; otherwise, Steps 3–5 are repeated.

### 3.2. Treatment of Constraints

In the OOP, there are two types of constraints: boundary constraints and technical limited constraint functions. The following is the treatment strategies of them.

#### 3.2.1. Boundary Constraints

Sometimes **u**_*i*,*G*_ is out of the range of search space. When it happens, it is necessary to replace this value to guarantee it is into its allowed range (12). Consider

(12) uij,G={xijL+rand·(xijU−xijL)   if  (uij,G<xijL) ,  or  (uij,G>xijU)uij,G, otherwise          i=1,2,…,NP;  j=1,2,…,D,

where *x*_*ij*_^*L*^ is the lower bound of *u*_*ij*,*G*_; *x*_*ij*_^*U*^ is the upper bound of *u*_*ij*,*G*_.

#### 3.2.2. Constraint Functions

Penalty function is one of the most effective methods to solve the evolutionary constraint optimization problem [21–25].

In this paper, the MINLP with constraints (13) is converted into an unconstrained MINLP by using a penalty function shown as (14). Consider

(13) min⁡f(x,y)s.t.gk(x,y)≤0,k=1,2,…,phl(x,y)=0,l=1,2,…,qxiL≤xi≤xiU,i=1,2,…,Dyk∈{0,1},k=1,2,…,m,

(14) min⁡  F(x,y)=f(x,y)+M ×[∑k=1pmax⁡⁡{0,gk(x,y)}2    +∑l=1qmax⁡⁡{0,|hl(x,y)−ε|}2],

where *x* = [*x*_1_,*x*_2_,…,*x*_*n*_]^*T*^ is a continuous vector; *y* = [*y*_1_,*y*_2_,…,*y*_*m*_]^*T*^ is a binary vector; *g*_*k*_(*x*, *y*) represents the *k*th inequality constraint; *h*_*l*_(*x*, *y*) represents the *l*th equality constraint; *F*(*x*, *y*) represents new objective function, which consists of two parts: the old objective function *f*(*x*, *y*) and a penalty function; *p* is the number of inequality constraints; *q* is the number of equality constraints; *ε* is a small positive constant, so that the *l*th equality constraint *h*_*l*_(*x*, *y*) = 0 is converted into the inequality constraint *h*_*l*_(*x*, *y*) − *ε* < 0. In addition, *M* is defined as penalty coefficient, which is a large positive constant, so that it imposes penalty on unfeasible solutions.

### 3.3. Conventional Technique for Binary Variables

In this paper, the {0, 1} binary variables represent on/off status of RO units, but the DE algorithm is only capable of handling continuous variables. Therefore, some real variables within the range of [0, 1] are used to represent the statuses of RO units in TSDE instead of these binary variables. When the objective function and the constraint functions are calculated, these real variables are rounded off to the nearest integer with (15). Consider

(15) $$\begin{array}{c} {\overset{-}{y}}_{i}=\text{INT}\left(y_{i}\right), \end{array}$$

where INT() is a function to convert a real number to an integer value by rounding off.

### 3.4. Two-Stage DE

The OOP of a large-scale parallel-unit SWRP desalination plant has such features: the permeate rate of each RO unit is huge; that is, the amount of freshwater generated by each RO unit is usually more than 10000 tons a day; the permeate rate changes within an allowable range, but this range is much smaller than its amount. So, when a RO unit's status changes, the TRC of the plant will change sharply. Usually, this change cannot be made up by adjusting the RO unit's permeate rate within its allowable range.

In this paper, a novel DE, TSDE, is proposed to solve the OOP. The TSDE is divided into two periods: Stage One and Stage Two. In Stage One, the permeate rate of each RO unit is supposed be a constant, which equals the median of its allowable range. A DE algorithm is used to compute the run/off status of each RO unit in this stage. When the DE is satisfying the stopping criterion, a preliminary scheduling will be worked out.

Then, the best 30 percent individuals in Stage One remain to Stage Two. The other 70 percent individuals in Stage Two are generated in the search space randomly (here, these two stages have the same population size NP). All these individuals are as the original values of another DE algorithm to evolute again in Stage Two. When the second DE stops, a final scheduling will be got.

## 4. Experimentation

For numerical experimentation, a large-scale parallel-unit SWRO desalination plant in Liuheng Island, China, which has the capacity of 100,000 m^3^ freshwater a day, is considered over a 24-hour time horizon. The basic parameters of this OOP are shown in Tables 1, 2, and 3.

Here, the length of individual *D* = 20; each individual consists of 20 variables. The first 8 variables are {0, 1} binary variables to represent the on/off statuses of 8 RO units. The next 8 variables are real values, which are the amounts of freshwater generated by 8 RO units. And the last 4 variables are real values to represent the supplied freshwater by 4 PFWTs.

The CDC of this plant is considered as a constant and ignored when calculating the TRC.

### 4.1. Parameters of TSDE

The DE's search ability for different problems depends on its parameters [26]. So, before it is working, these parameters must be tuned.

#### 4.1.1. The Maximum Number of Iterations *G*_*m*_ and the Population Size NP

Firstly, the effects of two important parameters, the maximum number of iterations *G*_*m*_ and the population size NP, on the search ability of DE for the OOP are explored. A basic DE is used to study the relationship, setting *F* = Rand[0.1 : 0.2], CR = Rand[0.7 : 0.9], where Rand[*a* : *b*] represents a uniformly distributed random value that ranges from *a* to *b*.

The different pairs {*G*_*m*_, NP} for DE are used to compute the solution of the OOP, and each algorithm is running 10 times. The results are listed in Table 4, in which the bold font is the best solution. It can be observed that the bigger these two parameters are, the stronger the search ability is.

It is worth noting that if *G*_*m*_ and NP are too big, the search speed of DE will decline sharply; and if the NP is small to a certain value, the algorithm is easy to fall into the locally optimal solution. Therefore, there is a compromise between the search speed and the accuracy when turning these two parameters.

#### 4.1.2. The Scale Factor *F* and Crossover Rate CR

The scale factor *F* and crossover rate CR are another two important parameters in DE; the search ability of DE is sensitive to them too. In order to find out the effects of *F* and CR on the search ability of DE in this problem, a sensitivity analysis of these two parameters is presented.

**Figure 3:**
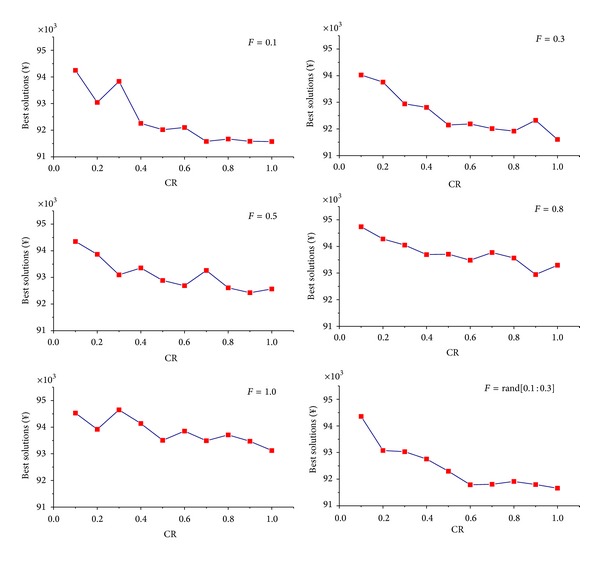
Analysis of CR in different *F*.

**Figure 4:**
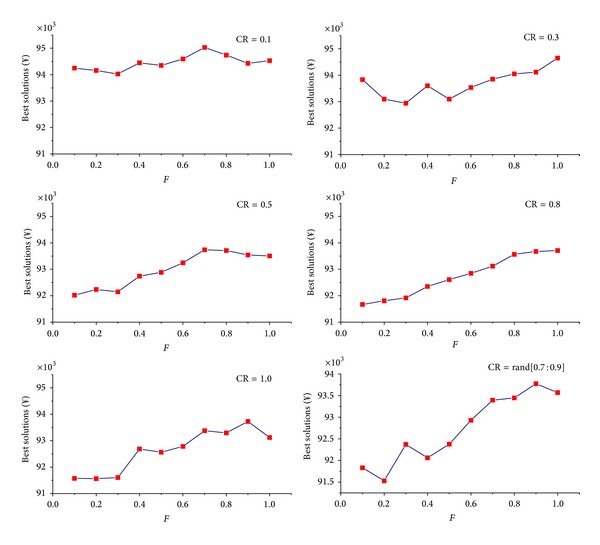
Analysis of *F* in different CR.

Usually the *F* is in the range of [0.0, 1.0] and CR is in the range of [0.0, 1.0] [15]. In order to compare the effects of different *F* and CR fairly, the same initial individuals are used to calculate the optimal values of this OOP under different *F* and CR. The population size NP = 100 and the maximum number of iterations *G*_*m*_ = 1000. A basic DE is used to do this job, and the results are shown in Figures 3 and 4.

In Figure 3, the effects of different CR on the search ability of DE when *F* = 0.1, 0.3, 0.5, 0.8, 1.0, and Rand[0.1 : 0.3], respectively, are explored, in which it is found that OOP gets its best solution when CR⩾0.7. In Figure 4, the effects of different *F* on the search ability of DE when CR = 0.1, 0.3, 0.5, 0.8, 1.0, and Rand[0.7 : 0.9], respectively, are studied, and it is found that OOP gets its minimal value when *F* ⩽ 0.3. So, we set *F* = Rand[0.1 : 0.3] and CR = Rand[0.7 : 0.9] for DE algorithm to compute the best solutions of OOP in the following sections.

#### 4.1.3. The Two Maximum Numbers of Iterations *G*_1_ and *G*_2_ of TSDE

As mentioned in the above sections, the TSDE algorithm is divided into two stages. The two maximum iteration numbers of these two stages, *G*_1_ and *G*_2_, must be determined before the TSDE working. Here, it is supposed that *G*_1_ + *G*_2_ = 1000, NP = 100, *F* = Rand[0.1 : 0.3], and CR = Rand[0.7 : 0.9], and each algorithm runs 10 times. The results are listed in Table 5, in which the bold font is the best solution. It is known that the TSDE gets its best solution when *G*_1_ = 300 and *G*_2_ = 700.

### 4.2. Pseudocode of TSDE

The pseudocode of TSDE is shown in Algorithm 1.

### 4.3. Comparative Study

#### 4.3.1. Comparison between TSDE and Other Global Minimizing Algorithms

In this paper, genetic algorithm (GA) and basic DE are chosen to compare the search ability with STDE for this problem. The main parameters of GA are set as follows: the maximum number of iterations *G*_*m*_ = 1000, the length of chromosome *L*_*c*_ = 20, mutation factor *F* = 0.6, and crossover rate CR = 0.1; the main parameters of basic DE: the maximum number of iterations *G*_*m*_ = 1000, the population size NP = 100, mutation factor *F* = Rand[0.1 : 0.3], and crossover rate CR = Rand[0.7 : 0.9]; the main parameters of TSDE: the maximum two numbers of iterations *G*_1_ = 300, *G*_2_ = 700, the population size NP = 100, mutation factor *F* = Rand[0.1 : 0.3], and crossover rate CR = Rand[0.7 : 0.9]. All algorithms are coded in MATLAB 7.0 and executed in HP desktop 6300 MT with Intel Core i5-3470 CPU @3.2 GHz and 4 GB RAM.

Each of algorithm runs 10 times, and the statistical results are listed in Table 6, in which the bond font are the best solution. The average searching quality of TSDE is better than the others. Moreover, the standard deviations by basic DE and TSDE are much smaller than GA.

#### 4.3.2. Comparison between Optimal Operation and Manual Operation

Finally, we compare the optimal operation with manual operation in the OOP. The scheduling strategy of manual operation is that all RO units are running until reaching the high amount limits of the PFWTs from time 1 to time 8 every day, when the time-of-use electricity price is the lowest. When the freshwater amount of each PFWT is at high limit, the corresponding RO units stop. At other time periods, the running status of each RO unit is only determined by the user's freshwater demand, and the time-of-use electricity price will no longer be considered. The optimal operation obeys the scheduling plan computed by TSDE.

**Figure 5:**
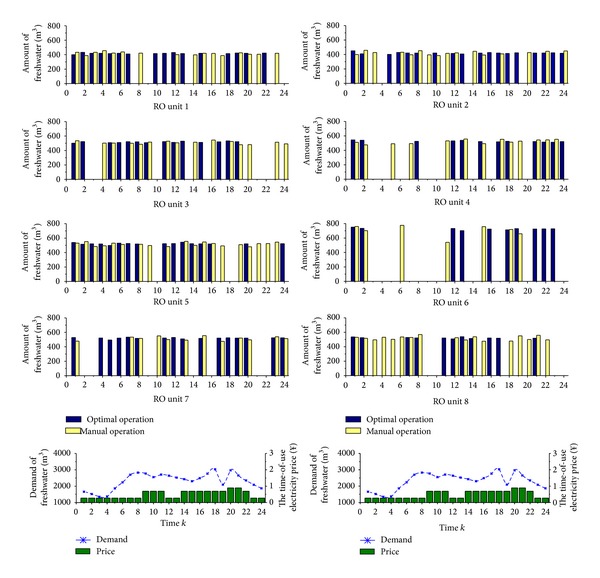
The comparison of capacity of each RO unit.

Figure 5 is the comparison of freshwater generation of each RO unit between optimal operation and manual operation. As Figure 5 shows, those RO units which have smaller freshwater generation capacity, such as Unit 1 and Unit 2, are almost running because of its smaller running cost. On the contrary, the RO unit which has large capacity (its running cost increases according to its capacity), such as Unit 6, is opened only when necessary. So, it has low utilized efficiency. The others which have medium capacity are working intermittently.

**Figure 6:**
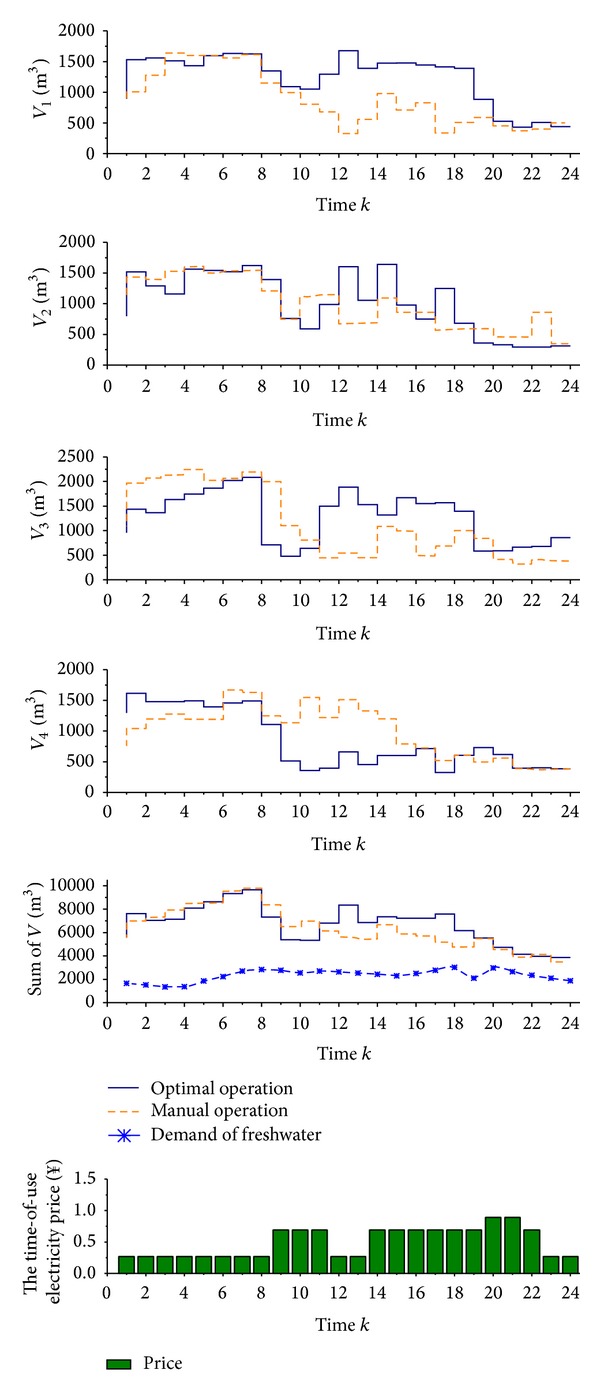
The comparison of capacity of each RO unit.

Figure 6 is the comparison of capacity of each PFWT. The sum of capacity of all PFWTs fluctuates with the profile of freshwater demand.

From Figure 6, it is observed as follows.

**Figure 7:**
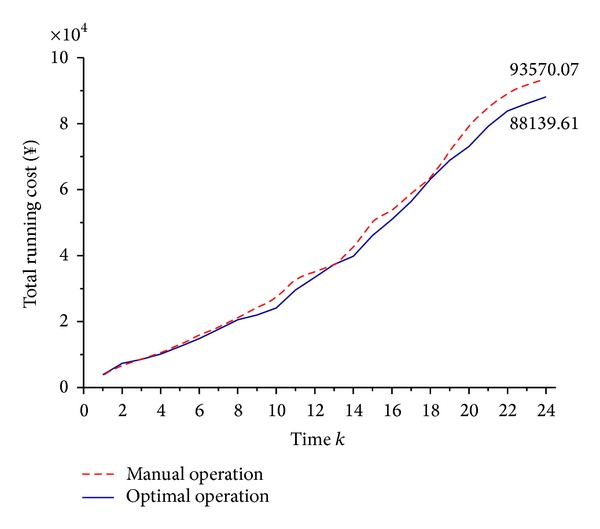
The comparison of TRC.

1. Both of these two schedules open the RO units to generate freshwater from time 1 to time 8, and the sum of freshwater reaches its peak at time 8;
2. the manual operation does not take the time-of-use electricity price as the cost factor of TRC after time 8, so even at the highest electricity price period, such as time 15, the sum of freshwater of PFWTs is still increasing. That is, the RO units are still opening at these time periods, so that the TRC under manual operation cannot get its optimal value;
3. the optimal operation takes full use of its advantage in global optimization, so at times 8, 13, and 18, the sum of generated freshwater reaches its local peaks before the time-of-use electricity price gets higher. Therefore, when the electricity price is higher, the storage of freshwater is used to satisfy user's demand, and the RO units will not be opened unless the storage of freshwater reaches its lower limit. In this way, the TRC of this plant under optimal operation is 5% lower than manual operation (Figure 7).

## 5. Conclusions

The OOP of large-scale parallel-unit SWRO desalination plant is modeled as a MINLP, in which binary-valued vectors indicate the on/off statsues of RO units and real-valued vectors indicate the amounts of freshwater generated by RO units and the amounts of supplied freshwater by PFWFs. The objective function of the OOP is the total running cost of the desalination plant, and the constraint functions include the technical limitations, the design requirements of each RO unit, and the freshwater demand of user. A novel DE, two-stage DE, is presented to solve this OOP, and the effects of its main parameters on the search ability are analyzed in this paper. Applying this TSDE to a 100,000 ton SWRO desalination plant in Liuheng Island, China, it is observed that the proposed TSDE can successfully improve the utilization rate of RO units to reduce the TRC.

## Figures and Tables

**Algorithm 1:**
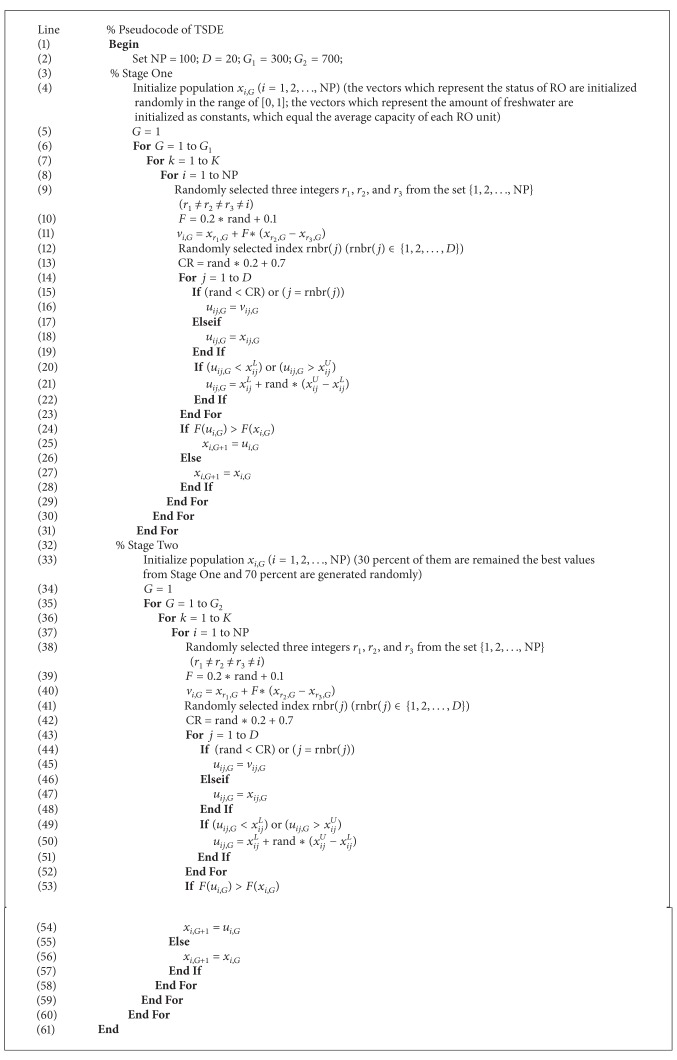
Pseudocode of TSDE.

**Table 1:** The basic parameters of OOP in Liuheng SWRO desalination plant.

Parameters	Sign	Value
The number of RO units	*n*	8
The number of PFWTs	*m*	4
The number of RO units which feed freshwater to each PFWT	*n*_1_	2
	*n*_2_	2
	*n*_3_	2
	*n*_4_	2
The upper limit of the permeate rate of RO Unit *i*, (m^3^/h)	*Q*_1,max⁡_	460
	*Q*_2,max⁡_	460
	*Q*_3,max⁡_	570
	*Q*_4,max⁡_	570
	*Q*_5,max⁡_	570
	*Q*_6,max⁡_	800
	*Q*_7,max⁡_	570
	*Q*_8,max⁡_	570
The lower limit of the permeate rate of RO Unit* i*, (m^3^/h)	*Q*_1,min⁡_	380
	*Q*_2,min⁡_	380
	*Q*_3,min⁡_	470
	*Q*_4,min⁡_	470
	*Q*_5,min⁡_	470
	*Q*_6,min⁡_	655
	*Q*_7,min⁡_	470
	*Q*_8,min⁡_	470
The initial value of PFWT (m^3^)	*V*_1,0_	340
	*V*_2,0_	340
	*V*_3,0_	340
	*V*_4,0_	340
The maximum capacity of PFWT *i*, (m^3^)	*V*_1,max⁡_	1680
	*V*_2,max⁡_	1680
	*V*_3,max⁡_	2280
	*V*_4,max⁡_	1680
The minimum capacity of PFWT *i*, (m^3^)	*V*_1,min⁡_	320
	*V*_2,min⁡_	320
	*V*_3,min⁡_	320
	*V*_4,min⁡_	320
The maintenance cost coefficient	*C*_1_	11.5
The repair and replacement fees	*C*_2_	155
The correlation coefficient of energy consumption and generated freshwater	*C*_3_	2.86

**Table 2:** The user's demands for freshwater *D*(*k*).

Time *k*	Demands for flash water (m^3^)	Time *k*	Demands for flash water (m^3^)
1	1660	13	2525
2	1520	14	2430
3	1345	15	2300
4	1370	16	2490
5	1865	17	2765
6	2235	18	3030
7	2700	19	3095
8	2830	20	2970
9	2770	21	2655
10	2555	22	2350
11	2705	23	2085
12	2640	24	1870

**Table 3:** The time-of-use electricity price *P*_*e*_(*k*).

Time *k*	1–8	9–11	12-13	14–19	20-21	22	23-24
Electricity price (*¥*/kWh)	0.27	0.69	0.27	0.69	0.89	0.69	0.27

**Table 4:** Analysis of NP and *G*_*m*_ of TSDE.

NP	*G*_*m*_	Best solution (*¥*)	Mean value (*¥*)	Std. dev.
200	250	89758	90376.7	351.6491
250	200	89662	90471.4	556.8511
334	150	89116	89666.7	410.5998
500	100	88822	89926.1	423.0399
625	80	88725	89508.5	451.2566
**1000**	**100**	**88429**	**88955.4**	**376.5983**

**Table 5:** Analysis of *G*_1_ and *G*_2_ of TSDE.

*G*_1_	*G*_2_	Best solution (*¥*)	Mean value (*¥*)	Std. dev.
**300**	**700**	**88140**	**88745.38**	**467.6048**
500	500	88464	88878.9	383.1357
700	300	88556	88850	166.2274
1000	0	88275	89052	489.2724

**Table 6:** Comparison of the best solutions of STDE with basic DE and GA (*¥*).

Algorithm	Best solution	Worst solution	Mean value	Std. dev.
GA	89680	91102	90556	622.9885
Basic DE	88429	89266	88955.4	376.5983
**TSDE**	**88140**	**89246**	**88791.59**	**391.3258**

## References

[1] Wade NM (2001). Distillation of plant development and cost update. *Desalination*.

[2] El-Zanati E, Eissa S (2004). Development of a locally designed and manufactured small-scale reverse osmosis desalination system. *Desalination*.

[3] Villafafila A, Mujtaba IM (2003). Fresh water by reverse osmosis based desalination: simulation and optimisation. *Desalination*.

[4] Marcovecchio MG, Aguirre PA, Scenna NJ (2005). Global optimal design of reverse osmosis networks for seawater desalination: modeling and algorithm. *Desalination*.

[5] Lu YY, Hu YD, Xu DM, Wu LY (2006). Optimum design of reverse osmosis seawater desalination system considering membrane cleaning and replacing. *Journal of Membrane Science*.

[6] Lu YY, Hu YD, Zhang XL, Wu LY, Liu QZ (2007). Optimum design of reverse osmosis system under different feed concentration and product specification. *Journal of Membrane Science*.

[7] Saif Y, Elkamel A, Pritzker M (2008). Optimal design of reverse-osmosis networks for wastewater treatment. *Chemical Engineering and Processing*.

[8] Sarkar P, Goswami D, Prabhakar S, Tewari PK (2008). Optimized design of a reverse osmosis system with a recycle. *Desalination*.

[9] Majali F, Ettouney H, Abdel-Jabbar N, Qiblawey H (2008). Design and operating characteristics of pilot scale reverse osmosis plants. *Desalination*.

[10] Verhuelsdonk M, Attenborough T, Lex O, Altmann T (2010). Design and optimization of seawater reverse osmosis desalination plants using special simulation software. *Desalination*.

[11] Sassi KM, Mujtaba IM (2013). Optimal operation of RO system with daily variation of freshwater demand and seawater temperature. *Computer and Chemical Engineering*.

[12] Lee YG, Gambier A, Badreddin E, Lee S, Yang DR, Kim JH (2009). Application of hybrid systems techniques for cleaning and replacement of a RO membrane. *Desalination*.

[13] Jing DW (2006). *Optimization Design of Reverse Osmosis System*.

[14] Storn R, Price K (1995). Differential evolution: a simple and efficient adaptive scheme for global optimization over continuous spaces.

[15] Storn R, Price K (1997). Differential evolution—a simple and efficient heuristic for global optimization over continuous spaces. *Journal of Global Optimization*.

[16] Storn R, Price K (1997). Differential evolution-a simple evolution strategy for fast optimization. *Dr. Dobb's Journal*.

[17] Storn R, Price K (2006). *Differential Evolution—A Simple and Efficient Adaptive Scheme for Global Optimization over Continuous Spaces*.

[18] Storn R, Price K, Lampinen J (2005). *Differential Evolution—A Practical Approach to Global Optimization*.

[19] Zhong YW, Wang LJ, Wang CY, Zhang H (2012). Multi-agent simulated annealing algorithm based on differential evolution algorithm. *International Journal of Bio-Inspired Computation*.

[20] Yang XS, Deb S (2012). Two-stage eagle strategy with differential evolution. *International Journal of Bio-Inspired Computation*.

[21] Carroll CW (1961). The created response surface technique for optimizing nonlinear restrained systems. *Operations Research*.

[22] Fiacoo AV, McCormick GP (1968). Extensions of SUMT for nonlinear programming: equality constraints and extrapolation. *Management Science*.

[23] Coello Coello CA (2002). Theoretical and numerical constraint-handling techniques used with evolutionary algorithms: a survey of the state of the art. *Computer Methods in Applied Mechanics and Engineering*.

[24] Huang F-Z, Wang L, He Q (2007). An effective co-evolutionary differential evolution for constrained optimization. *Applied Mathematics and Computation*.

[25] Mallipeddi R, Suganthan PN (2010). Ensemble of constraint handling techniques. *IEEE Transactions on Evolutionary Computation*.

[26] Ponsich A, Coello CAC (2011). Differential evolution performances for the solution of mixed-integer constrained process engineering problems. *Applied Soft Computing Journal*.

